# Peer Perspectives on Challenges Encountered During a Multi-Site Digital Mental Health Intervention Project

**DOI:** 10.1007/s10488-025-01441-2

**Published:** 2025-04-19

**Authors:** Biblia S. Cha, Elizabeth V. Eikey, Dana B. Mukamel, Kristy J. Palomares, Stephen M. Schueller, Dara H. Sorkin, Nicole A. Stadnick, Sarah Elizabeth Stoeckl, Kai Zheng, Margaret L. Schneider

**Affiliations:** 1 Department of General Internal Medicine, University of California, Irvine, CA USA; 2 Herbert Wertheim School of Public Health and Human Longevity Science, University of California, San Diego, CA USA; 3 The Design Lab, University of California, San Diego, CA USA; 4 Department of Public Health, University of California, Berkeley, CA USA; 5 Department of Informatics, University of California, Irvine, CA USA; 6 Department of Psychological Science, University of California, Irvine, CA USA; 7 Department of Psychiatry, University of California, San Diego, La Jolla, CA USA; 8 Dissemination and Implementation Science Center, UC San Diego Altman Clinical and Translational Research Institute, La Jolla, CA USA; 9 Child and Adolescent Services Research Center, San Diego, CA USA; 10 Joe C. Wen School of Population & Public Health, University of California, Irvine, CA USA; 11 School of Medicine, University of California, 100 Theory, Suite 120, Irvine, CA 92697 USA

**Keywords:** Peer support, Digital mental health, Mental health services, mHealth, Implementation

## Abstract

Peers are individuals with lived experience of mental health challenges trained to provide support to others with similar challenges. Help@Hand was a multi-site project that integrated peers into digital mental health intervention (DMHI) implementation. This study uses the Consolidated Framework for Implementation Research (CFIR) to frame challenges reported by peers when implementing DMHIs. Individuals leading the local peer workforce completed quarterly online surveys about perceived challenges to DMHI implementation. Biannual interviews probed for details on survey-reported challenges. 103 quarterly surveys and 39 bi-annual interviews were collected from key informants at 11 Help@Hand sites between Summer 2020 and Fall 2022. One challenge was tied directly to DMHIs; namely, device distribution. Several related to the Implementation Process, including challenges with recruiting qualified peers and integrating peers into DMHI implementations; communication and collaboration; and translation. Challenges in the Individual domain included unclear peer roles and multi-tasking across various projects. Inner Setting challenges included structural barriers to hiring peers, issues with communication and project management, and workforce turnover. Outer Setting challenges related to environmental technology readiness, COVID-19, unclear decision-making processes across the collaborative, and uneven communication between sites’ peers. Funding uncertainty bridged the Inner and Outer Settings. Using the CFIR model to frame challenges to DMHI implementation yielded useful lessons, especially when peers are engaged as partners in planning and implementation process. Successful implementation will be enhanced by ensuring adequate environmental readiness for tech-based interventions, clear role definition, streamlined peer hiring processes, and well-delineated lines of communication locally and across sites.

## Introduction

Mental health is a global health concern (Beeber, 2019; Greenberg et al., 2021; Walker et al., 2015), and a significant portion of individuals who need help do not receive treatment (Jorm et al., 2017; Merikangas et al., 2011; Substance Abuse and Mental Health Services Administration, 2020). Digital mental health interventions (DMHIs) are one promising strategy for addressing barriers to traditional in-person mental health services, especially accessibility and acceptability of mental health care (Lattie et al., 2022), such as concerns about privacy and stigma (Andrade et al., 2014; Reardon et al., 2017). DMHIs can include devices (such as smartphones, tablets, computers, and wearables) and software (such as mobile health apps, websites, and care management systems) that facilitate in-person or tele-health mental health treatment through digital means (Borghouts et al., 2021; Schueller et al., 2013). Encouragingly, studies indicate that some DMHIs have effectiveness comparable to or greater than traditional in-person therapy for improving symptoms (Carlbring et al., 2018; Karyotaki et al., 2017).

## Peers and DMHI Implementation

In the context of mental health care, peers are individuals with lived experience of mental health challenges who are in recovery and are trained to provide support to others with similar challenges (Fortuna et al., 2022). Studies indicate that peer support as an adjunct to mental health services can positively impact psychosocial outcomes (White et al., 2020) and that DMHIs facilitated by peers can be more effective in improving mental health symptoms compared to those without peer support (Werntz et al., 2023). Furthermore, studies have concluded that it is critical to engage mental health consumers to design “relevant, usable, and effective” digital health tools (Baker et al., 2014) and have documented advantages of engaging them early in the design process (Jonathan et al., 2021). Thus, involving peers with both DMHI design and implementation may be critical to DMHI success (Vargas et al., 2022). Engaging peers in the implementation processes for DMHIs can encourage and facilitate end-users’ uptake of the technology (e.g., Funnell et al., 2022; Ludlow et al., 2023; Pozuelo et al., 2023), and also benefit peers themselves in their recovery journey (Myers et al., 2021).

However, recent reviews have concluded that peers are rarely engaged by researchers and designers in collaborative implementation of mental health interventions (Åkerblom & Ness, 2023; Brotherdale et al., 2024). Efforts to integrate peer input specifically for DMHI development and implementation have faced various challenges, including time and documentation required for such efforts, the need to provide safe spaces for those involved in the various processes, distrust based on peers’ prior experiences of not feeling heard, and existing power dynamics between peers and others involved (Maathuis et al., 2020; Milton et al., 2021; Kruzan et al., 2021). Moreover, there is a dearth of studies that examine challenges to implementing DMHIs in real world service contexts as observed by peers themselves.

### The Current Study: Help@Hand

Help@Hand was a state-wide five-year initiative funded by the California Mental Health Services Act (MHSA) and centrally coordinated by the California Mental Health Services Administration (CalMHSA). Help@Hand was aligned with the overall goal of the MHSA to “expand and transform California’s behavioral health system” while “improving the quality of life for Californians living with mental health challenges” (Mental Health Services Act, 2020). Specifically, the Help@Hand project explored whether and how integrating DMHIs within local county/city mental healthcare systems could enhance access to mental health services, improve mental health service quality, and improve mental health outcomes, especially among underserved, unserved, and inappropriately served individuals (California Department of Health Care Services, 2023; Help@Hand, 2023; Sorkin et al., 2023). A key feature of the project was involving peers throughout the process of Help@Hand planning and implementation.

Analyses of the specific activities undertaken by peers during Help@Hand and the perceived outcomes of the integration of peers in Help@Hand demonstrated considerable variability across sites in terms of how and to what extent peers were actually involved in the planning and implementation of DMHIs (Cha et al., 2024; Schneider et al., Epub ahead of print). At one end of the spectrum, one county designed their own DMHI and involved peers in every step, including the design of the technology, implementation of the intervention, and delivery of digitally mediated peer counseling (Take My Hand™, n.d.). At the other end of the spectrum, a few of the Help@Hand sites never hired a staff member to oversee the integration of peers into the program. Among the sites that did engage at least one peer staff member, the average number of peer-led activities per quarter ranged from fewer than one to more than five (Cha et al., 2024). We have previously described the activities peers engaged with during Help@Hand (Cha et al., 2024), as well as the outcomes peers perceived resulted from being involved with DMHI implementation (Schneider et al., Epub ahead of print). The current study extends these findings by examining the range and scope of challenges reported by peers throughout the implementation of the Help@Hand program.

## The Consolidated Framework for Implementation Research and the Help@Hand Project

Theoretical frameworks are useful to understand factors that hinder or facilitate strategic development, dissemination, and uptake of DMHIs (Berkout & Sunal, 2023). The Consolidated Framework for Implementation Research (CFIR) is a framework commonly used to understand, predict, or explain facilitators and barriers to specific intervention implementation (Damschroder et al., 2022). Five primary domains (Innovation, Outer Setting, Inner Setting, Individuals, and Implementation Process) are used to group factors that can influence intervention implementation. The CFIR can be applied at any phase of implementation (pre-implementation, during implementation, or post-implementation), which allows for flexible adaptation and application for a wide range of interventions on varying timelines (Kirk et al., 2016).

In the current study, we applied the CFIR retrospectively to frame challenges that were reported by peers during the implementation of their local Help@Hand projects. This work builds on existing scholarship to examine challenges related to partnering with individuals with lived experience when designing DMHI implementations. We discuss the implications of our findings for others interested in implementing similar efforts in their local contexts.

## Methods

### Study Context

With 39 million residents, California is the most populous state in the US (U.S. Census Bureau, 2020). This study considers data from the following Help@Hand sites: City of Berkeley, Tri-City (a group of three cities: Pomona, Claremont, and La Verne), and Marin, Mono, Orange, Riverside, San Francisco, Santa Barbara, Tehama and San Mateo Counties. San Mateo County was counted as two sites because two Peer Leads supported two separate populations, resulting in a total of 11 sites.

The 11 sites included large metropolitan areas that were 100% urban as well as smaller regions that were 100% rural or had less than 9,000 residents (U.S. Census Bureau, n.d.). Funding, resources, and staffing capacity also varied widely across sites. With total funding for all project sites at over $101 million, specific sites received widely disparate amounts of funds ($26,000-$25 million). Each of the sites went through their own processes to recruit and hire their peer workforce, including a Peer Lead, who was a peer specifically assigned to implement the peer component of a site’s Help@Hand project who coordinated other peers hired at the site.

### Study Participants

Key informants, most of whom were Peer Leads, completed interviews and surveys across three years of the Help@Hand project. If a site Peer Lead was not available to participate in the survey and/or interview, data were collected from the Tech Lead (the overall Help@Hand project lead) or the MHSA Coordinator (coordinator for Mental Health Services Act-related programs) for the site. Data were collected from the same individual at each site as long as they remained available. The number of sites engaged in data collection was reduced from 11 to nine after Spring 2022 due to two sites ending their Help@Hand participation at that point.

### Data Collection

This study was conducted by an evaluation team from the University of California, Irvine. Quarterly online surveys were conducted to collect reports of peer experiences, including successes (as described in Cha et al., 2024) and challenges that occurred between September 2020 and December 2022. Four times during this period, interviews were conducted shortly after survey data collection (Table 1). This study was approved by the Institutional Review Board of the University of California, Irvine.

**Table 1:** Schedule of data collection

Quarter	2020		2021				2022			
	Sum	Fall	Win	Spring	Sum	Fall	Win	Spring	Sum	Fall
Number of sites	11	11	11	11	11	11	11	11	9	9
Number of surveys (Response Rate)	11 (100%)	11 (100%)	11 (100%)	10 (91%)	11 (100%)	11 (100%)	11 (100%)	9 (82%)	9 (100%)	9 (100%)
Number of Interviews (Response Rate)	9 (82%)	–	11 (100%)	–	10 (91%)	–	–	9 (82%)	–	–

#### Challenges Survey

Key informants were emailed a link to the online survey using REDCap (Research Electronic Data Capture), a secure, web-based software platform designed to support data capture for research studies (Harris et al., 2019). They were asked to consider the previous quarter in their responses, i.e., Winter (January-March), Spring (April-June), Summer (July-Sept), or Fall (October-December). Reminders were sent every three days until surveys were completed, up to a maximum of six reminders. Incomplete surveys were considered missing data one month after the initial survey invitation.

#### Challenges Interview

Interviews were conducted in English over the telephone or using Zoom audio. Key informants provided verbal consent. Interviews typically lasted between 30 min and 1 h. MLS, who has extensive training in interviewing and qualitative data analysis, conducted all but one interview. To encourage frank sharing, interviews were not audio-recorded, and the interviewer took extensive near-verbatim notes. Key informants were assured responses would only be reported in aggregate or as anonymous quotes. Quotes are presented only with year and interviewees’ role to further protect their privacy.

## Measures

### Challenges Survey

In Summer 2020, a survey was developed based on a rapid qualitative analysis of 22 semi-structured formative telephone interviews conducted with Help@Hand Peer Leads in Winter and Spring 2020 (Cha et al., 2024). During the formative interviews, Peer Leads were asked: (1) “How, if at all, has your [site] used peers to support Help@Hand in the last 2–3 months?” and (2) “How effective do you think the peers are in supporting Help@Hand?” An additional six survey items were added to the survey in Fall 2021 informed by a rapid qualitative analysis of the interviews conducted between Summer 2020 and Fall 2021. Survey items were presented as a checklist, and respondents were asked to check those challenges they had experienced in the previous quarter.

### Challenges Interviews

The purpose of the interviews was to elicit detailed information about challenges reported on surveys and to learn about new challenges that emerged over time. Key informants were first presented with a general open-ended question asking for a brief summary of the overall status of the Help@Hand project, a best practice in qualitative research interviewing (McGrath et al., 2019). The interviewer then asked the key informant to provide details regarding challenges reported on the survey (See Appendix 1 for interview guide). The interview ended with a general question about the respondent’s perception of how effective they thought peers were in supporting Help@Hand.

### Data Analysis

After data collection was concluded, four team members (BSC, MLS, KJP, SES) conducted an in-depth qualitative analysis of interviews to identify additional challenges that may not have emerged during the rapid qualitative analysis. Interviews were coded using ATLAS.ti 24.0.1 (ATLAS.ti, 2023). The coding team individually coded four interviews and then met to discuss primary and secondary coding categories, including creating new codes that differentiated from survey items. The team used this preliminary codebook to code another seven interviews, which were reviewed again as a team to discuss new codes and definitions. The remaining interviews were coded by the coding team and previously coded interviews were reviewed by the first author to ensure consistency of coding with the updated codebook. Finally, BSC and MLS reviewed coded interviews to identify quotes that elaborated on reported challenges. Using all the challenges that had been identified by surveys and the in-depth qualitative analysis, we computed the prevalence of each challenge (i.e., the proportion of sites reporting each challenge at least once, where the numerator was the number of sites that reported a challenge at least once, and the denominator was 11, the total number of sites).

Finally, to aid in the interpretation of the data, challenges were grouped according to CFIR domains. In the context of this study, we identified the “Innovation,” which is typically the product being implemented, as the DMHIs. The “Inner Setting,” which is the setting in which the Innovation is implemented, was identified as the mental health department which housed the Help@Hand project as well as the setting where peers were employed if the peer component was subcontracted out. The “Outer Setting” was comprised of CalMHSA, the collaborative partnerships among sites, relationships between sites and technology vendors, and the broader physical and social contexts outside the Inner Setting that exerted influence on DMHI implementation. The “Implementation Process” included the processes and activities that were required to get the DMHI up and running. Finally, the CFIR domain labeled “Individuals” was identified as the peers who were hired to support the DMHI implementation process.

## Results

### Study Participants

Twenty-one key informants completed 103 surveys and 39 interviews between Summer 2020 and Fall 2022. Seven of the 11 sites in the study completed 100% of the ten quarterly surveys. Surveys were completed by Peer Leads (79%), Tech Leads (17%), and Peer Lead Supervisors or MHSA Coordinators (4%). Interviews were completed by Peer Leads (80%) and Tech Leads (10%).

### Challenges

Challenges to implementing the peer component of Help@Hand were identified related to the Innovation, Individual, Inner and Outer Setting, and Implementation Process domains of the CFIR (see Table 2 for specific challenges and exemplary quotes). In addition, we identified one challenge that spanned the boundary of the Inner and Outer Settings. In the following sections, we expand on the nature of the challenges reported within each of the CFIR domains and provide additional quotations from the interviews to illustrate the depth and complexity of the sites’ experiences.

### Innovation Challenges

The Innovation domain of CFIR encompasses unique attributes of the program or intervention being studied. In keeping with the flexible strategies supported by the Help@Hand consortium, some of the Help@Hand sites chose to distribute digital devices to community members, and this program component engendered delays because of logistical issues, such as obtaining devices, installing software, or ensuring that devices met site specifications for data security. Nearly half (45%) of sites reported project delays related to distributing digital devices (e.g., cell phones, iPads) to the community. One Peer Lead shared, “We hit a snag because of needing to wait for higher ups to approve the budget and sign off, then we got the news of the chip shortage” (2021).

### Individual Challenges

Another CFIR domain highlights aspects of the people who are instrumental in implementing the intervention. Analysis of the survey and interview data revealed that Individual challenges in the Help@Hand project emerged in relation to the peers, who were involved in the DMHI implementation processes and whose perspectives were the focus of this study. One such challenge was a lack of clarity about the role of peers within the project, which was reported by about two-thirds of sites. One Peer Lead described this problem: “There could be better role discernment. Once the peers know what they are doing they really excel. We go through a cycle—ambiguity, clarity, ambiguity. Depending on where we are in that cycle it impacts their effectiveness” (2021). In addition, 82% sites reported that peers were required to divide their efforts among competing priorities, with limited time dedicated specifically to the Help@Hand projects. As another Peer Lead stated bluntly, “[Help@Hand] is just one of my jobs, and I have other things to do” (2020).

**Table 2:** Challenges to DMHI implementation observed by peers organized by CFIR domain and prevalence (%)

	Challenge	Quote	%
Innovation	Delays related to device distribution^b^	We outsourced our IT [information technology] stuff to a company called [vendor] […] We had to work with them, we went back and forth on what tablets we were going to do. We had CalMHSA looking into [tablets]. That took a little while. —Peer Lead	45
Individual	Peers were unclear about their role on the Help@Hand project^c^	What I can say is that if I am supposed to be taking the lead—I don’t know who is in charge. —Peer Lead	64
	Peers had to divide their time and effort among projects^b^	Looking at our staff– a lot of them are part timers so doing this outside of their scope of work is hard for them. —Tech Lead	82
Implementation Process	Challenges related to the recruitment of qualified peers^a^	The peer that we hired would have been fantastic if the job involved running a peer support group—but for the skill set we needed it was way off. It was a recruitment failure. —Tech Lead	91
	Peer input was solicited or offered but not integrated into decisions^b^	Peers want to have a voice in everything that is happening on this project, but sometimes the project needs to move ahead, and marketing information has gone out without feedback from the peers. —Peer Lead	27
	Challenges related to the flow of information between site and the Help@Hand collaborative^a^	There were times when our county monitor would tell us that there was a change and there was not enough time for us to implement it–information that wasn’t shared with us. —Peer Lead	82
	Challenges related to vendor collaborations^c^	I remember that even though we were having issues with [vendor], it was a difficult time because [the peers] were hired by [vendor], and we would be in meetings with negative talking about [vendor]. —Peer Lead	64
	Delays related to getting contracts finalized^b^	We are at a complete standstill. There are contract things that need to be done between the county and the [technology] vendor regarding information sharing and that is where it has been for the last several months. —Peer Lead	82
	Need for translation of program materials^a^	Since I don’t speak Spanish, when it comes to having translations, we have to pay someone to do our translations—that hinders us. —Tech Lead	64
Inner Setting	Challenges related to the hiring and/or onboarding of qualified peers^a^	“When you are hiring a peer, because mental illness has been criminalized by our society, they may have a hard time passing a background check. Peers may have a criminal background. —Peer Lead	91
	Challenges related to the dissemination of information within a site^a^	The last Help@Hand meeting I went to we had decided about [technology] and I was told in the last month that we had also decided on [technology]. The mental health manager is more involved than I am, and I did not know that we were doing two. —Peer Lead	91
	Peer workforce is too small^b^	We don’t have many peers so that is definitely a challenge. —Peer Lead	82
	Organizational, structural, and/or process challenges within a site^c^	There is not enough staffing.… During tech distribution, one person should be focused on the outreach. Then someone needs to distribute the devices, then someone needs to provide tech support. —Peer Lead	64
	Turnover among the peer workforce^a^	Our Peer Lead retired, and they did not make any arrangements for someone to take over her spot. —Tech Lead	55
Outer Setting	Lack of clarity regarding decision-making processes across the collaborative^a^	I think if you were to ask me whose decision it is to make on almost anything, I would not be able to tell you. Within the collaborative-I would not know who to direct you to. It seems unclear to me. —Peer Lead	73
	Challenges related to technology issues, including digital literacy, access to devices/internet^c^	One of the greatest barriers is having access. That was something that was overlooked in part of this project. They just assumed that everyone knows how to get on the internet. With the digital literacy training we found that there were so many people who could not even access the equipment. —Peer Lead	91
	COVID 19^c^	Because of the fact that we were reduced to social distancing, working from home, we had challenges with how people were going to do their job from home. —Peer Lead	82
	Challenges related to Peer Collaborative calls^c^	Most of [the peer collaborative calls] fall on the same day as a leadership meeting I have to attend […] When I do attend, 6 out of 10 are useful because you get to hear about what other counties are doing. But sometimes because I am so disconnected I don’t know what to take in. —Peer Lead	64
Inner/Outer Settings	Uncertainty about the sustainability of the program owing to funding-related issues^b^	The only thing I have concerns about is how the peers may feel about their limited time status. It will probably cause some anxiety and stress as we wind down with the project. Even though it was clear up front that the project is time limited and it is not going to be forever. I think I am already starting to sense with one of the peers worries about what is the job security beyond Help@Hand. —Peer Lead	64

*Notes* Prevalence was computed as a percent of the total sites reporting the challenge at least once. ^a^Derived from the initial formative interviews; ^b^Derived from the rapid qualitative analysis of post-survey interviews; ^c^Derived from in-depth qualitative analysis of post-survey interviews

### Implementation Process Challenges

Six of the challenges reported by sites can be characterized as aspects of the Implementation Process, or activities and strategies used to implement the DMHI. These specifically related to challenges to the coordination, collaboration, and communication between various entities, reflecting that the Help@Hand project was a state-funded initiative centrally coordinated by the funder (CalMHSA) that required local sites to recruit peers into their projects, integrate peer input, engage with outside vendors to bring DMHIs to their local communities, and reach out to community members. The challenges encountered when implementing the DMHI included difficulties with recruiting peers, integrating their input into Innovation-related decisions, communicating information between the sites and CalMHSA, working with DMHI vendors, negotiating contracts and translating materials.

Central to implementing the Innovation were the recruitment of peers and the integration of their input. Peers were envisioned as bringing a lived experience perspective to the planning and delivery of digital technology tools with the intent of increasing access to mental health services among underserved communities. Nearly all (91%) of the Help@Hand sites reported that recruiting peers qualified for these roles was a challenge. The unique combination of lived experience with mental health challenges plus expertise in digital technology made it difficult to find qualified individuals for the positions. This challenge was clearly described in an early interview: “The goal was to find a peer who represented the population we would be serving. Finding someone with lived experience […] that has technical knowledge was a real challenge. The skill set that we need is someone who is organized, comfortable with public speaking, can work somewhat independently, can coach around digital health and technology […] This is challenging in the payscale, and it is only 18 hours per week. It is hard to fill” (2020). In addition, because the peers were hired with the expectation that their input would be integrated into decisions that shaped DMHI implementation, key informants shared that it reduced peer effectiveness when peer input was not actually sought or used. This challenge was reported by 27% of sites, and was attributed to short timelines, cost of implementing recommendations (such as translation of apps into different languages), and lack of organizational readiness. It was articulated well by one Peer Lead: “Peers want to have a voice in everything that is happening on this project, but sometimes the project needs to move ahead, and marketing information has gone out without feedback from the peers” (2022).

Three of the Implementation Process challenges were related to collaborating with entities outside of the local departments. One was rooted in the lines of communication between the funding agency, CalMHSA, and the individual Help@Hand sites. Specifically, 82% of sites reported challenges related to the unclear or delayed flow of information from the funding agency to local sites. This challenge was partially related to staff turnover at CalMHSA, which resulted in sites having to engage with and adjust to new styles of communication and leadership. One Peer Lead shared, “We went from one person to another person and it took a while for us to understand the difference in style. Our project management leader came in guns blazing with a new organizational style—we were trying to switch gears” (2021). Another challenge reported by two-thirds of sites originated from the process of collaborating with technology vendors. In some cases, vendors backed out of the Help@Hand project after being chosen. In other cases, there were communication issues with vendors that caused delays. One Peer Lead shared that, “Disappointingly we had an app ready that we wanted to pilot and we had to stop the hiring process because the developers of [technology] backed out and said they could not work with us during COVID-19” (2020). Another Peer Lead described challenges related to frequent back and forth communications that delayed multiple launches and ultimately caused an indefinite postponement: “We are trying to go into the pilot stage for the past couple of months. We have been at the point of going to launch it three separate times but because of issues for the developer or getting consent we have had to push it three different times and now we don’t have a date, because there has been so much back and forth. The current status is that we need to get it started ASAP and we don’t have the tools to get that going” (2021). The majority of sites (82%) also reported experiencing delays in contracting, whether with CalMHSA or a vendor. The novelty of working with digital technology is one factor that accounted for these contracting delays, which translated into periods of time when peer efforts had to be redirected into other tasks. For example, one Peer Lead stated, “We are almost there but there has been some contractual stuff around privacy and sharing of information. Our [site] is really conservative, they are very careful about sharing information […] That has been the situation for a while—at least a month” (2021). Finally, two-thirds of sites pointed out their inability to effectively reach members of their target population owing to a lack of both marketing and intervention materials that were in languages appropriate for the communities they were seeking to reach, as many of the technologies were limited in their language choices and project funds had not been allocated for translation at the beginning of the project. One Peer Lead reported, “It took months to get a Spanish landing page for the project, so we were putting off reaching out to Spanish populations” (2021).

### Inner Setting Challenges

Among the five Inner Setting challenges that were identified, three were related to structural issues that affected staffing. Specifically, sites reported difficulty with hiring and retaining peers, and observed that their sites’ peer workforce was not robust enough to meet the demands of the project. Survey data indicated that nearly all (91%) sites reported that hiring qualified peers posed a challenge for their project, while interviews revealed that hiring difficulties often were attributed to organizational policies that made it difficult to specifically hire peers who had lived experience with mental health challenges. For example, one Peer Lead stated, “As a project we can all agree what peer means, but then counties, when hiring for these roles, there is a lot of work that needs to be done, because local [Human Resources] departments are seeing this information, and we can’t ask these questions because of [Health Insurance Portability and Accountability Act], or it can been seen as discriminatory” (2021).

The majority (82%) of sites reported that their peer workforce was too small. Key informants voiced concerns about the discrepancies they observed between project goals and allocated resources, especially when many of the peer workforce were part-time employees or had other full-time responsibilities. One Peer Lead was vocal about the needs at the local level: “The Help@Hand project requires more than what is made known. For example, digital literacy training, the technical support hours, the device procurement. We have added these on even though we were only supposed to roll out [technologies]. It would have been better to make it known to our peers that there might be other elements that could be added. There was an expectation that the project was more limited, but it needed the support elements to make it work” (2021). In addition, over half (55%) of sites reported that peer staff turnover was a challenge. Whether due to mis-matched expectations of the work, disability leave, retirement, or assignment to other positions, recurring changes in peer staffing made continuity of projects difficult. For example, one site reported that “Our Peer Lead retired and they did not make any arrangements for someone to take over her spot. We are trying to figure out how that position will be replaced—still figuring it out” (2021).

Another two challenges in the Inner Setting were related to local project management. These challenges included inefficient communication within local sites (reported by 91% of sites) and difficulties with the local processes or management structure (reported by 64% of sites). One Peer Lead noted that “there are people making decisions and I don’t know about them. I am getting it kind of third hand. I don’t know how involved peers were in any other aspect” (2021). Another Peer Lead shared that: “In my term, coming up on 2 years, I had 3 different leaders that I have worked under. Every time I have had to rework different processes to have them understand what is going on. They always have their own ideas about how things should run, that slows down the process” (2022).

### Outer Setting Challenges

Four of the challenges reported resided in the larger context outside the control of the agency or department implementing the Help@Hand project. These were either related to communications that originated outside of the local site or to broader issues that influenced DMHI implementation. First, a lack of clarity in decision-making processes across the collaborative was reported by approximately three-quarters of sites. Reflecting the project’s external funding and coordination by CalMHSA, one Peer Lead stated: “There is a lot of misunderstanding around who is making what decisions and how that information is disseminated. There is a lot of confusion about how it is being funded or why we can’t do certain things” (2020).

About two-thirds of sites also reported difficulties in peer-specific cross-site communication. At the beginning of the Help@Hand project, the CalMHSA peer project manager established a monthly call to facilitate a learning community among the peers, but after this individual’s departure, key informants reported that calls were not effective for many reasons. For sites unable to attend these meetings for logistic reasons, project personnel could not learn from activities and strategies shared by other site peers. One Tech Lead shared, “I get the sense that the peers feel like they are not heard. Since I am not on the peer calls, I don’t know why that is happening…there is some disconnect there” (2020). Key informants also indicated that the level of energy and participation in the peer calls shifted over time, with several monthly calls being cancelled, resulting in decreasing educational value for participants. As one Peer Lead shared, “You hear a lot of dead air on those [peer call] meetings. A lot of peers have fear around speaking and there is not an equal amount of peer activity at each location […] I don’t know that they are priming the pump enough for as much participation. The peer manager is new, she is trying to find her feet. I think that they could use some structure. It is flat” (2021).

A prominent Outer Setting challenge to implementing the peer component was the COVID-19 pandemic, mentioned by 82% of sites. The pandemic posed project challenges such as the cancellation of in-person meetings and activities, diversion of attention and funds away from the Help@Hand project and hiring freezes or personnel shifts to pandemic-related activities. The pandemic left sites uncertain about how to move their projects forward and both project personnel and community members moved to remote communications and interactions. For example, one Peer Lead shared: “Initially a lot of the trainings with potential consumers and clients and members were going to be face-to-face—those didn’t happen because of the COVID situation” (2020).

Another Outer Setting challenge experienced by virtually all sites (91%) was the lack of community proficiency with and access to technology. This included low digital literacy and lack of access to devices or internet connection. While these issues were at times characteristic of specific groups, such as older adults, the frequency of the challenge indicated that difficulty accessing or using technology was fairly pervasive throughout communities. After the COVID-19 pandemic moved most activities online, sites saw how this shift accentuated the ‘digital divide.’ As stated by one Peer Lead: “There is a digital divide, and we are realizing that now when we are sheltering at home and some people don’t even have the Zoom capabilities. Some people don’t have the finances or the ability to have smart phones” (2020). A Tech Lead reported that this required peers themselves be very tech savvy in order to assist community members with varying levels of digital literacy: “What I learned is that you really have to know what you are doing, because every situation is different technologically- what internet browser they are using, what device they are using. You really have to know it all to get them able to use the technology” (2020). Sites reported having to provide support even among young people, who were assumed to be more technologically adept, as noted by one Peer Lead: “A lot of young people had not used Zoom or Google hangouts at all before. So even trying to get on an initial call with them was difficult” (2020).

### Challenge Spanning the Inner and Outer Setting

Key informants also identified uncertainty about future project funding as a challenge that spanned the Inner and Outer Setting:. This uncertainty led to some peers departing the project and/or sites being reluctant to hire new peers. 64% of sites reported uncertainty about future peer funding, which was initially provided through the state-funded project. While some cited specific concerns about peer employment, others ruminated on the implications of nearing the end of the Help@Hand contract, when funded activities would cease unless funded by other sources within the site department. Early in the project, one Peer Lead shared, “Contractually we will be done in 2024. We talked about what if we have a [group] where we train them on digital literacy and then they could be ambassadors […] but if the program does not extend beyond 2024, it does not make sense to put that time into it” (2021).

## Discussion

We examined the challenges that emerged as sites worked to engage peers in shaping implementation of technology-based interventions for improving access to mental health services. Previously, we described the activities that peers contributed to during Help@Hand (Cha et al., 2024) and the outcomes that peers observed from their involvement with DMHI implementation (Schneider et al., Epub ahead of print). This study extended those findings by documenting challenges reported by peers throughout Help@Hand implementation. By using the CFIR as a lens through which to organize the different challenges, we found that sites encountered challenges residing in a range of domains. Our analysis of these challenges addresses a critical gap in the literature for programs seeking to engage peers as partners in the mission to improve access to mental health services with the support of digital technology (Åkerblom & Ness, 2023; Brotherdale et al., 2024), and provides insights and recommendations for future research and practice.

### Factors Affecting the Peer Workforce

The Help@Hand project aimed to integrate peers at all levels of the project; yet, peers identified numerous factors affecting the capacity of the peer workforce to realize this intention.

### Implementation Process-Related Challenges

First, recruiting qualified peers was a critical challenge for many sites, even as California passed Senate Bill 803 to become the 49th U.S. state to permit Medicaid billing for peer support services and implement a peer certification program the early phases of the Help@Hand project (California State Legislature, 2020). While the legislation recognized and affirmed the value of peers as partners in mental health care delivery, it was not specifically designed with the intent to involve peers in the process of designing the program implementations like Help@Hand. Thus, sites found it difficult to find individuals who brought the unique combination of mental health-related life experience and technical and language skills sought for these positions. Prior studies have highlighted the importance of gathering input from individuals with lived experience of mental health challenges, as well as other organizational staff, before recruiting and hiring peers into the mental health workforce to enhance the likelihood of effective and sustainable efforts (Bellamy et al., 2017). These studies have suggested that organizations can improve peer recruitment by underscoring the desirability of their life experiences rather than educational or certification requirements, and preparing human resources personnel to address peer disclosures of mental health experiences during the recruitment and hiring stages (Bellamy et al., 2017). While these studies do not specifically address the technical skills that peers in the Help@Hand project often used, they indicate that future similar projects will likely need to bolster outreach efforts and build in sufficient time and organizational partnerships to reach qualified candidates. For agencies that do not have a critical mass of peers in-house, subcontracting with an existing peer organization may help to minimize the recruitment challenge, though studies conducted in other disciplines indicate that care is needed to ensure that subcontracted individuals are informed of and have buy-in regarding their employer’s organizational knowledge, culture, and communications (Willging et al., 2016).

Another important challenge was integrating peer input into local decision-making (reported by approximately a quarter of sites). Organizations commonly struggle in their efforts to shift structural and cultural factors to integrate peers into the workforce (Mancini, 2018), and studies have documented challenges to integrating peer input into mental health interventions (van der Meer et al., 2021; Mulvale et al., 2019; Milton et al., 2021; Kruzan et al., 2021; Maathuis et al., 2020). Our findings indicate that integrating peers into DMHI implementation involves efforts beyond stating an aspirational goal; it requires sufficient time, staffing, resources, and shifts in both individual norms and organizational culture. We highlight the comprehensive nature of these shifts as others have found that full integration is critical to avoid appending peers as tokenistic additions to a team (Kilpatrick et al., 2017).

#### Challenges with Peer Roles

Another key challenge to the Help@Hand implementation was unclear role definition for peers, a problem that did not improve over the Help@Hand project period. Unclear role definition has been identified as one of the most prominent barriers to the effective integration of peers into mental health care-related implementations (Mutschler et al., 2022; Vandewalle et al., 2016). One reason is that no standard description of peer services currently exists, and terms to describe peers vary based on contexts and types of services provided (Gaiser et al., 2021). In the context of the Help@Hand project, the variation in projects across the multiple sites led to peers being involved in different tasks and activities. Furthermore, after the departure of the individual hired by the project managers to coordinate the peers across the collaborative, local program managers had less centralized direction from the collaborative. The resulting ambiguity regarding peer roles and responsibilities may have been reinforced or amplified by the pressure that peers reported from competing job demands. Lack of role clarity impacts peers themselves and also leaves supervisors uncertain about how to train or evaluate the peers (Cabral et al., 2014; Mancini, 2018). Our findings reinforce the necessity to provide clear roles and responsibilities for peer staff to support their integration and success.

Based on our study, we recommend that individuals or teams planning and implementing programs that involve peers outline clear peer staff responsibilities and roles, and maintain fidelity to the roles throughout the implementation process, with space to be flexible based on program requirements or components that may change. Formalized organizational charts and continued dialogue across both peer and non-peer teams can be helpful to understand, clarify, revisit, and reinforce roles. These recommendations, which are consistent with strategies used to reduce tensions regarding role clarity and to help peers feel their roles are valued and taken seriously, include having supervisors acknowledge and take responsibility for lack of clarity, allowing for on-going discussions about peer role negotiation with supervisors, and having supervisors both assign and explicitly explain rationale for tasks (Stefancic et al., 2021). Additional potential strategies suggested in the literature for how implementation leaders can navigate the challenge of unclear roles for their peer staff include ensuring peers remain in roles that authentically leverage their lived experiences, embedding peer work within organizational policies and procedures, championing peer work at all organizational levels (both “bottom-up” and “top-down” support), and providing ongoing training both for peers and non-peer staff to ensure clarity of peer roles and to increase understanding and value of peers’ work (Byrne et al., 2022). While standardizing peer certification to promote standard levels of care provision may also encourage standardization of peer roles (Gaiser et al., 2021), we note this would not specifically address the diversity of projects implemented and activities engaged in by peers across the Help@Hand sites.

### Multiple Factors Affected the Peer Workforce

Sites also experienced difficulties related to the hiring and onboarding of qualified peers, peer staff turnover, and a peer workforce that was too small to meet the demands of the project, which is consistent with experiences reported in prior studies (Walker & Bryant, 2013). Among the many expectations that were placed on the Help@Hand peers, the most consistent was that they would facilitate the dissemination of the chosen technology to the community of interest. This critical role was assigned to peers in recognition of the substantial evidence that the effectiveness of DMHIs is limited by the extent to which consumers engage with the technology (Baños et al., 2022) and that engagement is enhanced by interpersonally facilitated support for the DMHI (Bourghouts et al., 2021). The challenges to maintaining a sufficiently robust peer workforce were therefore a major hindrance to realizing the potential of peers in program implementation.

Our study found that multiple factors in multiple domains ultimately led to Peer Leads reporting that their peer workforce was too small to effectively carry out necessary project activities. The outcome of both Implementation Process difficulties (i.e., recruiting peers with the unique combination of lived experience and ability to work with digital technology) and Inner Setting challenges (i.e., limited organizational resources and culture to support and retain peers over time) was an overall dissatisfaction with peer workforce size.

While the turnover rates experienced across the Help@Hand project reflect the general challenges of employing individuals in the process of mental health recovery (Walker & Bryant, 2013), studies suggest that peer workforce retention can be improved by ensuring structural support to peers transitioning into employment, including outlining clear roles and providing adequate and consistent supervision (Bellamy et al., 2017). Organizations can also prepare their human resources departments to address legal requirements for accommodations and ensure ongoing training for both peer and non-peer staff on recovery-oriented principles to enhance organizational readiness at all levels for the integration of the peer workforce (Bellamy et al., 2017). For sites reporting ongoing shortages in their peer workforce, we point readers back to the recruitment-related strategies described earlier but also suggest that there were lost opportunities for cross-site learning in the Help@Hand project. While there were sites with an established and robust workforce who could provide guidance and advice to other sites regarding structural, recruitment/hiring, and project-related factors that facilitated the hiring and retention of their peer workforce, the breakdown of the cross-site collaborative peer calls ultimately impeded this type of information- or strategy-sharing.

Our data corroborate studies indicating that programs engaging peers to support dissemination of a DMHI should proactively plan with the human resources department to provide more positions specifically for peers, streamline the hiring process, and budget for a workforce large enough to handle the work (Ahmed et al., 2012, 2015; Davidson et al., 2012; Mancini, 2018). In addition, sites would benefit from proactively planning to provide accommodations for peers experience setbacks in their recovery journey (Moll et al., 2009). An amorphous and dynamic organizational structure was also identified as an underlying issue affecting the peer workforce, and was highlighted especially for sites that did not previously have peers as a core part of their workforce. These sites were likely faced with limited organizational readiness and capacity to hire and integrate peers into their existing staff and practices (Mladenova, 2022), whereas sites who reported fewer challenges may have been operating based on a recovery-oriented model of mental health care (Salzer, 2010).

#### Technology Access and Digital Literacy

The focus on digital technology in the Help@Hand project uncovered additional challenges that were observed by the peers. First, lack of technology access and digital literacy was a challenge in the Outer Setting that was reported by nearly all sites. Digital literacy and technology access have been shown to vary widely by demographics (Atske & Perrin, 2021; Vogels, 2021). 16% of working-age American adults report low digital literacy (U.S. Department of Education, 2018), and several Help@Hand sites specifically sought to reach older adults and immigrant communities; populations that report lower rates of technology access and digital literacy compared to younger and native-born counterparts (Kakulla, 2024; U.S. Department of Education, 2018). In addition, partially due to the COVID pandemic, the program had to overcome the conundrum of how to reach individuals who were not digitally literate, did not have access to the internet, and/or did own devices, when the only available channel of communication was through digital technology. A subset of sites chose to distribute devices to members of the community to address their low rates of device ownership which introduced a new set of challenges, including selecting and obtaining the appropriate device and devising a plan for onboarding community members to the technology.

In confronting these challenges, the peers demonstrated the added value of their lived experience as they adapted their outreach efforts to meet the community members’ needs by delivering education in basic digital literacy and even orienting them to digital devices (Cha et al., 2024). Doing so aligns with prior research indicating that individuals experiencing barriers to engaging DMHIs benefit from ongoing technical assistance and facilitated support (Borghouts et al., 2022). It is important to note that for peers to be effective in this role, they themselves require training in digital technology, both initially and on an ongoing basis (Cha et al., 2024).

#### Delays in Peer Workflow

Throughout the Help@Hand project, peers reported project delays, many of which were rooted in the technological aspects of the project. Studies have shown health app implementations are often delayed by various misconceptions of the complex process of developing, launching, and maintaining a well-designed and sustainable mobile app, such as underestimating the effort and costs to design, build, test, and launch an app (Mannino et al., 2023). Peers in our study reported additional delays related to contract execution and negotiations with technology vendors over issues of data ownership, privacy, confidentiality, and sharing. Many peers used these waiting periods to develop outreach materials and deliver community digital literacy education (Cha et al., 2024), but over time, the delays (reported by 82% of sites) detracted from the overall effectiveness of the DMHIs. Local agencies disseminating DMHIs would be well advised to execute contracts with technology vendors early and to plan ahead for how best to deploy staff during likely delays.

Translation of DMHI content and promotional materials into priority languages for local communities was another implementation challenge, in part because funds had not been allocated for translation at the project outset. This challenge was made more difficult by virtue of the wide variety in language needs across the sites, and variations in preferred dialect or vocabulary. As vendors were reluctant and/or lacked the resources to provide digital technologies in multiple languages, several sites independently located digital products already available in the needed languages. Sites’ willingness to proactively find linguistically matched products highlights the critical importance of both planning outreach and providing DMHIs in languages appropriate for local communities. Such efforts are supported by evidence of meta-analyses concluding that interventions tailored for specific cultures and client native languages are significantly more effective than no treatment, treatment as usual, or unadapted interventions (Griner & Smith, 2006; Kalibatseva & Leong, 2014). Agencies considering DMHI implementation should assess local linguistic needs and plan staffing, time, and budgets accordingly.

Finally, the COVID-19 pandemic emerged as a prominent challenge in the Outer Setting. Studies indicate outbreak-related delays are not limited to COVID-19, such as a youth DMHI program that faced similar implementation hurdles as those experienced by Help@Hand sites due to meningococcus and measles outbreaks (Goodyear-Smith et al., 2021). Interestingly, because Help@Hand was a digital intervention, the pandemic accelerated deployment of some technologies that ultimately facilitated the program (e.g., rapid dissemination of video conferencing capabilities) (Dalle Torre, 2021).

#### The Critical Role of Clear Communication

Effective knowledge sharing is of critical importance for project and organizational success, and a common challenge that is not unique to Help@Hand (Almeida & Soares, 2014). However, the project’s need for internal, external, and cross-site communications made information flow a particularly challenging feature, spanning multiple domains (Implementation Process, Inner and Outer Settings). Information flow from CalMHSA to sites about budgetary guidance, assistance with executing contracts, and support for material translation was often delayed and unclear, leaving sites confused and unsure of how to proceed. In addition, sites reporting difficulties with intra-agency communication observed that peer activities were impeded by the poor flow of information within their local agency. Furthermore, sites struggled to adjust when the project experienced several changes in management, staffing and direction during the start-up phases (https://helpathandca.org/project-updates/reports/helphand-evaluation-reports/). The multiple changes in project management complicated communications and led to a lack of clarity in decision-making processes for sites. Difficulties that were reported with the cross-site peer collaborative calls may have been a downstream effect of the changes in project management and staffing. Our data suggest that peers might have continued to collaborate and learn from each other if the collaborative calls had been opened to more staff members at the individual sites, continuously led by a CalMHSA Peer Coordinator, and structured to encourage sharing of experiences across all sites. Our findings emphasize the need to proactively establish and maintain clear avenues of information sharing across the various project components for complex endeavors like Help@Hand.

### Influences of Funding Sources on Local Projects

The one challenge that crossed the Inner and Outer Settings (uncertainty about funding) was a product of the particular funding model for Help@Hand. As a state-funded project, Help@Hand was subject to oversight by the Mental Health Services Oversight and Accountability Commission (MHSOAC) of CalMHSA (Help@Hand, 2023). Moreover, as a publicly funded and time-limited project, Help@Hand was subject to certain regulatory and fiduciary restrictions. The uncertain funding situation introduced a reluctance to invest in building a stable peer workforce infrastructure. Future projects seeking to integrate peers into DMHI projects may benefit from establishing a sustainability plan up front to impart some sense of stability to peers hired to support this effort. Our findings also support prior recommendations for how to address the public health funding gap, including development of a public health infrastructure fund to support state and local agencies in their efforts to provide core public health capabilities (DeSalvo et al., 2019).

### Limitations

Our study methods and findings have several limitations. First, data were analyzed in three waves (i.e., formative interviews, rapid analysis of the early interviews, and in-depth coding of all interviews), resulting in some challenges being added over the course of the study. Second, interviews were not audio-recorded to encourage frank sharing during the interviews. Third, one reported challenge (funding uncertainty) did not fall neatly into any of the five CFIR domains, which is an acknowledged limitation of using the CFIR (Damschroeder et al., 2022). To address this discrepancy, we allowed for the funding challenge to be characterized as a “bridging factor,” a construct defined in other implementation models (Moullin et al., 2019).

## Conclusion

We synthesized challenges observed by peers during implementation of the Help@Hand project across 11 California sites. As DMHIs increase in availability and popularity, it is paramount for those involved in providing mental health services to strategically plan for how to consider perspectives of peers involved in implementation efforts (Lattie et al., 2022). Our analysis addresses a gap in the literature regarding peer perspectives on challenges to local DMHI implementation. Future research can further study factors that help or hinder the integration of peers into such implementations. Findings can also be used by sites planning similar projects and processes involving peers to consider the CFIR domains and potential challenges relevant to their respective local settings for optimal planning and implementation.

## Appendix 1

### Interview Guide

“Thank you for taking the time to talk with me again. I would like to remind you that I will not share your individual responses with anyone. We will only report and share what we learn from you after it has been combined with information from other counties/cities. There are no right or wrong answers. You are the expert. We are here to understand your perspectives and experiences. Do you have any questions before we begin?”

“Great! Let’s start with some general questions about your county’s/city’s utilization of Peers to assist with onboarding clients or users to Help@Hand. For reference, these questions refer to the time period that includes (previous three months).”

A. “I am going to ask you some specific questions that build on the information you provided in the survey, but before I do that, I would just like to get a sense overall of how things are going in your (county/city), especially with regards to the Peer component of Help@Hand. Can you give me an overall assessment?” 
B. “In your survey, you indicated that during the (quarter) of (year) the Peers:
C. “Your survey also indicated that (county/city) experienced the following challenges to involving Peers in Help@Hand during this time period:
D. “You also reported that (county/city) has had the following successes that have resultedfrom the Peer component of Help@Hand.
E. “What changes, if any, would you recommend for the Peer component of H@H movingforward?”
F. “How effective do you think the Peers are currently in supporting Help@Hand?” Why or why not?
G. Have you had a chance to look at the (Year) Evaluation Report produced by the team atUCI?
  a. If so, how, if at all, did it inform your Help@Hand programming?

Anything else?
